# The Hip Fracture Surgery in Elderly Patients (HIPELD) study: protocol for a randomized, multicenter controlled trial evaluating the effect of xenon on postoperative delirium in older patients undergoing hip fracture surgery

**DOI:** 10.1186/1745-6215-13-180

**Published:** 2012-09-27

**Authors:** Mark Coburn, Robert D Sanders, Mervyn Maze, Rolf Rossaint

**Affiliations:** 1 Department of Anaesthesiology, University Hospital Aachen, RWTH, Aachen, Germany; 2 Department of Anaesthetics, Intensive Care and Pain Medicine, Imperial College London, London, UK; 3 Department of Anaesthesia and Perioperative Care, University of California, San Francisco, USA

**Keywords:** Hip fracture, Postoperative delirium, Xenon

## Abstract

**Background:**

Strategies to protect the brain from postoperative delirium (POD) after hip fracture are urgently needed. The development of delirium often is associated with the loss of independence, poor functional recovery, and increased morbidity, as well as increases in length of hospital stay, discharges to nursing facilities, and healthcare costs. We hypothesize that xenon may reduce the burden of POD, (i) by avoiding the need to provide anesthesia with a drug that targets the γ-amino-butyric acid (GABA)_A_ receptor and (ii) through beneficial anesthetic and organ-protective effects.

**Methods and design:**

An international, multicenter, phase 2, prospective, randomized, blinded, parallel group and controlled trial to evaluate the incidence of POD, diagnosed with the Confusion Assessment Method (CAM), in older patients undergoing hip fracture surgery under general anesthesia with xenon or sevoflurane, for a period of 4 days post surgery (primary outcome) is planned. Secondary objectives are to compare the incidence of POD between xenon and sevoflurane, to evaluate the incidence of POD from day 5 post surgery until discharge from hospital, to determine the time to first POD diagnosis, to evaluate the duration of POD, to evaluate the evolution of the physiological status of the patients in the postoperative period, to evaluate the recovery parameters, to collect preliminary data to evaluate the economical impact of POD in the postoperative period and to collect safety data. Patients are eligible if they are older aged (≥ 75 years) and assigned to a planned hip fracture surgery within 48 h after the hip fracture. Furthermore, patients need to be willing and able to complete the requirements of this study including the signature of the written informed consent. A total of 256 randomized patients in the 10 participating centers will be recruited, that is, 128 randomized patients in each of the 2 study groups (receiving either xenon or sevoflurane).

**Trial registration:**

EudraCT Identifier: 2009-017153-35; ClinicalTrials.gov Identifier: NCT01199276

## Background

Postoperative delirium (POD) is an acute confusional state associated with changes in consciousness, arousal level and cognitive status characterized by inattention within 30 days after an operation [1] and occurs in 15 to 53% of older patients [1,2] particularly in those with hip fractures [1,3]. We have recently conducted a meta-analysis showing that delirium associated with hip fracture doubles the hazard ratio of death (HR 2.3 95% CI 1.4 to 3.9) [1]. Furthermore the development of delirium often initiates a cascade of events culminating in the loss of independence, poor functional recovery, and increased morbidity, as well as increases in length of hospital stay, discharges to nursing facilities, and healthcare costs [1-6].

The pathogenesis of delirium is poorly understood though Sanders has proposed that delirium can be considered as a ‘cognitive disintegration’ whereby the integrated neural function of the brain is broken down producing a varied range of symptoms focused on consciousness, arousal and memory [7]. Within the proposed framework various non-modifiable patient risk factors are hypothesized to contribute to reduced network connectivity (a surrogate for integration) at baseline, for example, age [7]. Modifiable risk factors (including inflammation and sedative drugs) act to further decrease network integration in the brain through altering the balance of neural transmission predominantly through increases in inhibitory tone mediated by γ-amino-butyric acid (GABA) signaling in the brain [7]. A prediction of this theory is that avoidance of GABAergic drugs, that include the majority of anesthetic (for example, sevoflurane or propofol) and sedative agents, would reduce the burden of postoperative and intensive care delirium.

In parallel, research showing multiple beneficial effects of the non-GABAergic anesthetic agent xenon has been published. As an inert, noble gas, xenon is not metabolized by the body but yet exerts myriad biological effects, the most notable being anesthesia and organ protection [8-18]. Xenon is thought to produce anesthesia through targeting either excitatory *N*-methyl-d-aspartate or two-pore-domain-potassium channels but not GABA_A_ receptors [8,9,13]. Xenon anesthesia is rapid onset, cadiostable and xenon is not thought to disturb autoregulation of organ blood flow [8,9,13]. Furthermore, xenon protects the brain, heart and kidney from diverse toxic insults including ischemia [8,9,13,18]. Xenon exerts a diverse array of neuroprotective effects (for example, induction of B-cell lymphoma-extra large (Bcl-xl), phosphorylated cAMP response element binding protein (pCREB) and hypoxia-inducible factor (HIF)-1α cell survival proteins) [11,12,18] and importantly synergizes with other neuroprotective strategies (for example, hypothermia and α2 agonists) [10,11,19].

We therefore hypothesize that xenon may reduce the burden of POD (i) by avoiding the need to provide anesthesia with a drug that targets the GABA_A_ receptor and (ii) through beneficial anesthetic and organ-protective effects. Based on our hypotheses we designed a multicenter European randomized controlled trial comparing xenon with sevoflurane anesthesia.

Our working hypotheses are: (1) xenon may contribute to a lower incidence of POD as compared to sevoflurane, within 4 days post surgery; (2) xenon may contribute to reduce postoperative organ dysfunction; and (3) xenon may reduce healthcare costs associated with POD.

## Methods and design

The design of the study was approved by the clinical ethical review committee (Clinical Ethical Review Committee; Medical Faculty; RWTH Aachen; EK 050/10) and by the local ethical review committees of the participating centers and by the competent authorities of the five participating countries prior to patient recruitment.

### Study centers

This trial was designed to include the following ten investigational centers: Department of Anaesthesiology, University Hospital Aachen, Aachen, Germany; Department of Anaesthesia, Imperial College London & Imperial College NHS Trust, London, UK; Department of Anaesthesia and Surgical Care, Hospital Clínico Universitario de Valencia, Valencia, Spain; Department Modul of Research in Anaesthesia, IRCCS Rizzoli Orthopaedic Institute, Bologna, Italy; Department of Anaesthesia and Surgical Intensive Care, Groupe Hospitalier Cochin Saint Vincent de Paul, Paris, France; Department of Anaesthesia, Centre Hospitalier et Universitaire de Grenoble, Hôpital Michallon, La Tronche, France; Department of Anaesthesia, Centre Hospitalier Régional Universitaire de Montpellier, Hôpital Lapeyronie, Montpellier, France; Department of Anaesthesia and Intensive Care Medicine, Centre Hospitalier et Universitaire de Toulouse, Hôpital Rangueil, Toulouse, France; Department of Anaesthesia, University Hospital Düsseldorf, Düsseldorf, Germany; Department of Anaesthesia, Emergency and Intensive Care Medicine, Klinikum Mutterhaus der Borromäerinnen gGmbH, Trier, Germany.

### Methodology

This phase 2 study is an international, multicenter, prospective, randomized, blinded, parallel group and controlled trial.

### Objectives

The primary objective of this study is to evaluate the incidence of POD, diagnosed with the Confusion Assessment Method (CAM; see Additional file 1) [20], in older patients undergoing hip fracture surgery under general anesthesia with xenon or sevoflurane, for a period of 4 days post surgery. The secondary objectives of this study are: to evaluate the incidence of POD from day 5 post surgery until discharge from hospital; to determine the time to first POD diagnosis; to evaluate the duration of POD; to evaluate the evolution of the physiological status of the patients in the postoperative period; to evaluate the recovery parameters; to collect preliminary data to evaluate the economical impact of POD in the postoperative period; and to collect safety data.

### Number of patients

A total of 256 patients will be included in this clinical trial. After having received local ethics committee and national competent authority approvals, patients are being recruited. Each patient who accepts to participate in the study will sign the written informed consent before performing any study specific procedure.

### Inclusion and exclusion criteria

The criteria for inclusion are: older patient (≥ 75 years); patient with planned hip fracture surgery within 48 h after the hip fracture; patient willing and able to complete the requirements of this study, including the signature of the written informed consent.

The criteria for exclusion are: patient suffering from multiple fractures, pelvic fractures proximal, pathological fractures, femur fractures (that is, fractures of the middle or distal femur); disabling neuropsychiatric disorders (severe dementia, Alzheimer’s disease, schizophrenia, depression); brain trauma within 12 months prior to selection, history of stroke with residuals; patient suffering from delirium (CAM diagnosis) at selection; patient who cannot complete the preoperative mental tests (CAM and/or Mini-Mental State Examination (MMSE)) of this clinical trial; patient with MMSE score < 24 at selection; patient known to susceptible to malignant hyperthermia; patient with elevated intracranial pressure; patient with a risk of high oxygen demand; patient with recent (less than 1 month) or ongoing myocardial infarction/damage; patient with severe cardiac failure or patient with severe impaired left ventricular systolic function; patient with known severe lung and/or airway disease, or severe chronic respiratory insufficiency, or a sustained homecare oxygen therapy; contraindication (serious illness or medical conditions) for general anesthesia; known allergy or hypersensitivity to any drugs administered during this clinical trial; previous participation in this clinical trial; participation in another clinical trial within 4 weeks prior to selection, or concurrent treatment with any other experimental drugs; history of alcohol or drug abuse or psychiatric disorders that would impair the understanding of the necessary information or render medically or legally unable to give written informed consent.

### Investigational plan and treatments

**Figure 1:**
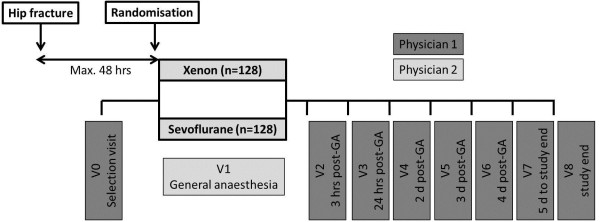
**Study flow chart of the Hip Fracture Surgery in Elderly Patients (HIPELD) study.** V = visit; d = day; hrs = hours, GA = general anesthesia.

Eligible patients will be randomly assigned to one of the two study groups (group A: xenon 60% (55% to 65%; 1 minimal alveolar concentration (MAC)) in oxygen (FiO_2_ = 0.35 to 0.45) or group B: sevoflurane 1.1% to 1.4% (1 MAC) in oxygen (FiO_2_ = 0.35 to 0.45) and medical air). Throughout the study, there will be two teams of physicians involved in the patient’s follow-up (see Figure 1).

Physician 1 will perform visits 0, 2, 3, 4, 5, 6, 7 and 8, which includes selection of patients, follow-up visits and study end visit. Physician 1 will be kept blinded regarding the natures and doses of all study drugs administered throughout the study (Table 1 and Figure 1).

**Table 1:** Study evaluations

	**Visit 0**^**a**^**(selection)**	**Visit 1**^**b**^ (**GA)**	**Visit 2**^**a**^**(3 h post GA)**	**Visit 3**^**a**^**(day 1)**		**Visit 4**^**a**^ (**day 2)**		**Visit 5**^**a**^ (**day 3)**		**Visit 6**^**a**^ (**day 4)**		**Visit 7**^**a**^ (**day 5 to D)**		**Visit 8**^**a**^ (**study end)**
				**A**	**B**	**A**	**B**	**A**	**B**	**A**	**B**	**A**	**B**	
Signed informed consent	X													
Selection criteria	X													
Demographic data	X													
Vital signs	X	X	X	X		X		X		X		X		
Medical and surgical history	X													
Concomitant diseases	X													
Concomitant medication	X	X	X	X	X	X	X	X	X	X	X	X	X	X
MMSE evaluation	X													
Randomization			X											
General anesthesia			X											
Blood sample	X			X		X		X		X		According to investigator’s judgment		
CAM evaluation	X		X	X	X	X	X	X	X	X	X	X	X	
Pain VAS evaluation	X	X	X	X		X		X		X				
Verbal Rating Scale for Nausea		X	X	X										
SOFA	X			X		X		X		X		According to investigator’s judgment		
Recovery parameters		X												
Adverse events		X	X	X	X	X	X	X	X	X	X	X	X	X

A represents 10:00 am ± 30 minutes; B represents 6:00 pm ± 30 minutes; day 5 to D is post-surgery day 5 until discharge from hospital (or at maximum 28 days post surgery), or withdrawal from the study.

^a^Physician 1; ^b^Physician 2.

CAM = Confusion Assessment Method; GA = general anesthesia; MMSE = Mini-Mental State Examination; SOFA = Sequential Organ Failure Assessment; VAS = visual analog scale.

Physician 2 will perform visit 1, which includes randomization and surgical procedure under general anesthesia.

### Pre-anesthetic assessment (visit 0, physician 1)

Within 48 h before their general anesthesia, cognitive function of each patient will be assessed with the MMSE, the presence of delirium will be diagnosed with the CAM, and the Sequential Organ Failure Assessment (SOFA) score ( Additional file 2: Table S1) [21,22]and level of pain on a 100-mm visual analog scale (VAS) will be assessed. Moreover, a 12-lead electrocardiogram (ECG) will be performed and a blood sample will be collected to assess laboratory parameters. Premedication for general anesthesia will be avoided before surgery, and no premedication impairing cognitive function will be administered on the day of the surgery. Of note, all investigators have been trained to use the CAM and all other test used in this trial.

### Surgical procedure under general anesthesia: randomization (visit 1, physician 2)

Patients must be on fasting state the day of the surgery, at least 6 h prior to anesthesia for solids and 3 h for liquids. Patients will be monitored continuously, and the corresponding measurements will be recorded every 5 minutes, for the following standard safety parameters: non-invasive blood pressure measurements (NIBP; that is, systolic and diastolic blood pressures), ECG monitoring, transcutaneous measurements of arterial oxygen (O_2_) saturation and end-tidal carbon dioxide (CO_2_) concentration, from the pre-anesthetic period to the end of general anesthesia. Body temperature will be monitored continuously. Patients will be equipped with a bispectral index (BIS) monitor for the depth of anesthesia, as well as with a monitoring device for the neuromuscular relaxation on their skin covering the adductor pollicis muscle. Physician 2 will take into account the neuromuscular relaxation monitoring to determine (i) the appropriate time for the patient’s endotracheal intubation, (ii) needs of repeated bolus injection of cis-atracurium during the maintenance period and (iii) appropriate time for the anesthesia discontinuation.

A registration number in sequential numerical order in each center separately will be assigned to each eligible patient. Before induction, physician 2 will open the individual randomization envelope allocated to the patient in order to prepare all equipment required and study drugs needed during the general anesthesia. Prior to the study start, one randomization list has been pre-established on computer by the sponsor.

### Induction of anesthesia

Preoxygenation will be performed using oxygen (FiO_2_ = 1.0) in all patients.

Balanced anesthesia will be induced by the combination of intravenous injection of propofol administered at 1 to 2 mg/kg by bolus titration; intravenous injection of remifentanil administered at 0.5 μg/kg via infusion pump continuously; and for tracheal intubation, non-depolarizing neuromuscular blocking agents, that is, intravenous injection of cis-atracurium will be administered at 0.1 mg/kg once the depth of anesthesia is considered sufficient (40 ≤BIS Index ≤60). The internal cuff pressure measurements will be recorded every 5.

### Maintenance of anesthesia

The administration of xenon or sevoflurane (according to the randomized study group assigned to the patient) will be started in the automatic mode (Felix DualTM workstation; Air Liquide, Paris, France) to reach a target concentration of 60% (55% to 65%) of xenon, or 1.1% to 1.4% of sevoflurane. About 10 minutes should be sufficient to reach this target concentration. This 10-minute period will be covered with a total intravenous propofol infusion at 0.05 to 0.15 mg/kg/min.

Then, the administration of propofol will be stopped. Simultaneously the xenon or sevoflurane will be administered in the economic mode (Felix DualTM workstation) for all patients to maintain the following target concentration according to randomized study group assigned to the patient: group A: xenon 60% (55% to 65%) (1 MAC) in oxygen (FiO_2_ = 0.35 to 0.45), group B: sevoflurane 1.1% to 1.4% (1 MAC) in oxygen (FiO_2_ = 0.35 to 0.45) and medical air.

Whatever their randomized study group assigned, patients will receive intravenous injection of remifentanil via infusion pump at an initial rate of 0.15 μg/kg/min, then titrated to the clinical needs. In addition intravenous bolus injections of cis-atracurium at 0.025 mg/kg will be administered according to physician 2’s judgment.

### Monitoring throughout the anesthesia

Systolic blood pressure should be kept ≥ 100 mmHg (or ≥ 60 mmHg for mean arterial blood pressure), and a relative variation > 20% from baseline values has to be avoided by administering rescue medication. The ECG will be monitored continuously and heart rate should be kept between 60 and 100 beats per minute. The type and duration of any new spontaneous arrhythmias should be recorded as adverse events. Ventilation will be adjusted to maintain an end-tidal CO_2_ level between 36 and 45 mmHg. Body temperature will be maintained between 36.0°C and 37.0°C. Depth of anesthesia will be continuously monitored with BIS VISTATM monitoring system (Covidien, Dublin, Ireland) and should be kept in a range between 40 and 60 to maintain a sufficient depth of anesthesia. Within the last 45 minutes before the expected skin closure, no additional cis-atracurium injection shall be administered, in order to ensure sufficient spontaneous ventilation prior to extubation. In addition, administration of acetylcholinesterase inhibitors should be avoided.

### Rescue treatments

If autonomic activity (defined as sweating, salivation, flushing), somatic signs (defined as movement and swallowing), or hemodynamic signs (defined as a change in systolic blood pressure or heart rate more than 20% from baseline in the absence of hypovolaemia) become evident corrective treatments will be administered according to physician 2’s judgment.

Antihypertensive and inotropic agents are allowed if the heart rate or blood pressures indicate their necessary use. Appropriate blood and/or fluid replacement strategy is to be used according to physician 2’s judgment. Analgesia, including additional locoregional analgesia, will also be administered according to physician 2’s judgment.

### Wake up

At 10 minutes before the expected end of all surgical procedures, the inhaled anesthetic agents will be reduced to 0.5 MAC. After the effective skin closure, the Train of Four (TOF) test will be performed to exclude existing neuromuscular relaxation. From this timepoint, adequate spontaneous ventilation has to be ensured and end-tidal carbon dioxide level is allowed to increase up to 50 mmHg. Recovery parameters, namely time to open eyes, time to react on verbal command, time to extubation and time to spatial orientation, are to be recorded.

### Post-anesthesia care unit (PACU)

The patients will be transferred to the PACU (if necessary, patients will be discharged directly to intermediate care or intensive care units) for continuous vital signs monitoring (NIBP, ECG and transcutaneous measurements of arterial oxygen saturation. Every 15 minutes, the vital signs and Aldrete score will be recorded until patients are considered ready to discharge from the PACU, that is, when their Aldrete score reaches a value ≥ 9 ( Additional file 3: Table S2) [23]. Postoperative nausea and vomiting (PONV) and blood loss will be assessed. The timepoint of this last evaluation of the Aldrete score will be considered as the end time of the recovery period, that is, the time to readiness to be discharged from the PACU.

### Postoperative pain management

Once the patient is able to evaluate his/her pain on a 100-mm VAS, postoperative analgesia (including additional locoregional analgesia) will be administered according to the local procedures of each participating center, in order to maintain a VAS pain score ≤ 30 mm. The administration of oral or intravenous non-steroidal anti-inflammatory drugs (NSAIDs), as well as opioids in the regional anesthesia, will be avoided.

### PONV management

Once the patient is able to evaluate his/her nausea on a 11-point verbal rating scale (VRS, Additional file 4) (with 0 corresponding to no nausea and 10 corresponding to nausea as bad as it could be) [24], Dexamethasone 8 mg and/or a 5HT3-antagonist (such as ondansetron 4 mg administered every 6 h if necessary) will be administered if the patient is retching or vomiting or if his/her VRS for nausea is > 3; see Additional file 4.

### Postoperative blood and/or fluid loss management

The blood and/or fluid replacement strategy is to be used according to physician 2’s judgment.

### Follow-up visits (visits 2 to 7, physician 1)

All randomized patients will be followed up from 3 h to discharge from the hospital (or at maximum 28 days post surgery), or withdrawal from the study. The 4 days following the surgery, CAM, VAS, SOFA and laboratory results, including platelets count, serum hemoglobin, hematocrit, leucocytes, aspartate aminotransferase (AST), alanine aminotransferase (ALT), γ- glutamyltransferase, total bilirubin, serum creatinine and urea concentrations, troponin I or troponin T, C-reactive protein, serum sodium and potassium and serum glucose concentration will be recorded. From day 5 onwards, SOFA and laboratory results become optional. Vital signs, concomitant medications, adverse events and serious adverse events are obtained in all study visits. Details of the study evaluations performed are presented in Table 1.

### Evaluation criteria

The primary efficacy criterion is POD diagnosed via CAM at least once within 4 days post surgery.

The secondary efficacy criteria include: (1) POD diagnosed with the CAM from day 5 post surgery to discharge from hospital; (2) SOFA from day 1 to day 4 post surgery; (3) recovery parameters; (4) economic parameters, which are addressed to evaluate the economical impact of POD in the postoperative period from the hospital perspective; (5) safety: adverse events and serious adverse events, and laboratory parameters.

### Safety evaluations

All adverse events (AEs) occurring during the whole study period will be recorded by the investigators. Physician 1 will record all AEs apart from those occurring on the day of the surgery, between the time of admission of the patients in the operating room to the time of discharge of the patients from the PACU. The information recorded in the case report forms for each AE experienced by the patients throughout their whole study period will include the nature of the events, their onset date and time, their end date and time, their severity, the corrective treatment(s) started if applicable, the outcome of the events and their relationship to the investigational inhaled gas and to the intravenous line applied separately as assessed by the investigator who records the AE. Physician 1 will record the relationships of each AE described to the investigational inhaled gas and to the intravenous line applied separately without knowing the nature of the study drugs administered.

### Economic parameters

Time to readiness to be discharged from the operating room, effective times of admission and discharge from the operating room, onset and end times of the general anesthesia and surgical procedure (onset and end times of induction, time of intubation, onset and end times of the administration of the study treatments, time of skin incision and end of wound dressing, time of extubation), total drugs (xenon or sevoflurane) consumption per patient, are to be recorded by Physician 2, as well as the type of hip fracture surgery performed (such as total hip replacement, hemiarthroplasty of the hip, dynamic hip screw, cemented or not cemented).

### Statistical methods

The main analysis of the primary efficacy criterion will correspond to the primary objective of the study which is to evaluate the incidence of POD, diagnosed via CAM, in older patients undergoing hip fracture surgery under general anesthesia with xenon or sevoflurane, for a period of 4 days post-surgery. All data collected will be tabulated descriptively by group on the intention to treat (ITT) dataset (which will be composed of all randomized patients). The number of patients having being diagnosed POD with the CAM at least once throughout their study participation from visit 2 to visit 6 will be calculated in each group, and this number of patients will be divided by the total number of participating patients of the corresponding group. This ratio which represents the rate of POD diagnosed within 4 days post surgery will be compared between both groups, using the Pearson’s *χ*^2^ test and its calculation provided by the FREQ procedure of the current version of the SAS software (SAS, Cary, NC, USA), on the ITT dataset.

Additional analyses of the primary efficacy criterion as time elapsed between the onset of the maintenance period of the general anesthesia and the first POD diagnosis with the CAM post surgery (expressed in full number of started hours) will be tabulated by group, as both quantitative and categorical data, in the subgroup of patients for whom POD is diagnosed at least once within 4 days post surgery. Time to first POD diagnosis with the CAM post surgery will also be described globally by group, on the ITT dataset, using Kaplan-Meier estimates to display the probability of survival and of being diagnosed POD. Survival times will be censored at the time of the patients’ last contact (in the subgroup of patients for whom POD is never diagnosed within their trial participation post surgery) or at the time of the first POD diagnosis with the CAM (in the subgroup of patients for whom POD is diagnosed at least once within their trial participation post surgery). If relevant, times to first POD diagnosis with the CAM post surgery will be compared between both groups with the Log rank test.

### Sample size calculation

We assume an expected rate of 30.0% of POD diagnosed with the CAM at least once within 4 days post surgery on sevoflurane, see Table 2[3,25-32]. We hypothesize a reduction of 50% to a rate of 15.0% of POD on xenon. The type I error is set to be α = 0.05 (two-sided conditions) and the power (1 - β) equals 0.80. Taking the above-mentioned facts into consideration, the sample size calculation results in 121 patients per group (the estimation is based on the two-group *χ*^2^ test of equal proportions). With an expected 5.0% dropout rate over 4 days post surgery, 256 patients will be randomized in this trial, that is, 128 patients in the xenon and 128 patients in the sevoflurane group. The sample size calculation was performed using nQuery Advisor® Version 6.01 (Statistical Solutions, Saugus, MA, USA). 

**Table 2:** Incidence of postoperative delirium

**Author**	**Study type**	**Patients**	**Age (years)**	**Method**	**Incidence**	**Comments**
Marcantonio E, 2001 [26]	RCT	64	>65	CAM	50%	Total (n = 126)
Marcantonio E, 2002 [27]	PCS	122	>65	CAM	40%	Subanalysis of [25]
Marcantonio E, 2002 [28]	PCS	126	>65	CAM	41%	Subanalysis of [25]
Furlaneto M, 2006 [3]	PCS	106	>65	CAM	29.1%	
Zakriya K, 2002 [29]	PCS	168	>65	CAM	28%	
Zakriya K, 2004 [30]	PCS	92	>65	CAM	28%	
Sharma P, 2005 [31]	PCS	47	>56	CAM	36%	
Galanakis P 2001 [32]	PCS	37	>60	CAM	40.5%	

This table is an excerpt from studies assessing delirium with the CAM. Based on these data the power for this study has been calculated.

CAM = Confusion Assessment Method; PCS = prospective cohort study; RCT = randomized controlled trial.

### Study monitoring, audit and inspection

The study will be monitored regularly (visits and telephone monitoring) by Air Liquide Santé International personnel or any company appointed by the latter. During the monitoring visits, the clinical research associate (CRA) will verify the consistency of the data recorded on the case report forms with the source documents (patient’s medical file, nurse’s chart, and so on). The CRA will also verify the management of therapeutic batches, the presence and completeness of the investigator file and general study compliance with good clinical practice guidelines. An on-site audit may be requested and performed by the sponsor or designee personnel at any time.

## Discussion

To date, 121 patients have been randomized (July 2012) in this phase 2 study. Inclusion of patients is approximately 50% slower than expected. For this reason the recruitment period of this trial was extended from the end of 2012 to the end of 2013. Furthermore, three new sites will be selected. Approximately two out of ten screened patients have been included so far. This is due to the strict inclusion and exclusion criteria especially cognitive impairment (MMSE < 24), dementia or depression. However, in this phase 2 study we need to keep above-mentioned inclusion and exclusion criteria to be able to show efficacy. We are awaiting the results of this trial to anticipate a phase 3/4 trial, which would have different inclusion and exclusion criteria including patients with reduced capacity. The primary objective of this study is to evaluate the incidence of postoperative delirium, diagnosed with the CAM, in older patients undergoing hip fracture surgery under general anesthesia with xenon or sevoflurane, for a period of 4 days post surgery. Postoperative delirium occurs predominantly in the first 4 days following hip fracture surgery [25], during this period it declines rapidly (peak on day 1, and on day 4). Thereafter it plateaus. Delirium occurring after 4 days is unlikely to be due to the anesthetic technique; therefore we only included the first 4 days for the primary endpoint. Furthermore the CAM is validated for use in older patients [33]. A big effort was made to design this study in s double blinded way and to keep the non-blinded bias as low as possible. Throughout the study, there will be two teams of physicians involved in the patient’s follow-up. Physician 1 will perform the visits, which include selection of patients, follow-up visits and study end visit. Physician 1 will be kept blinded regarding the natures and doses of all study drugs administered throughout the study. Physician 2 will perform randomization and surgical procedure under general anesthesia. However, physician 2 who is performing anesthesia is not blinded due to technical reasons applying xenon anesthesia. However, the bias of physician 2, who is aware of the randomization allocation, is kept minimal. Physician 2 is not involved in the assessment of the outcome parameters. Furthermore, the case report forms are strictly separated and kept locked so physician 2 is not able to access the case report forms of physician 1 and vice versa.

## Trial status

Patients are currently being recruited in all ten centers. The study was initiated in September 2010 and the predicted study completion date is December 2013. To date, 121 patients have been randomized (July 2012).

## Competing interests

MC, RDS, MM and RR received lecture and consultant fees from Air Liquide Santé International, a company interested in developing clinical applications for medical gases, including xenon. MC, RDS, MM and RR form the Scientific Committee of this study. RR and MC are involved as coordinating investigator and coinvestigator.

## Authors’ contributions

The Scientific Committee defines (in collaboration with the sponsor) and reviews the protocol. MC and RDS drafted the manuscript with input from MM and RR. All authors read and approved the final manuscript.

## Authors’ information

The HIPELD Investigators

Belda J: Department of Anaesthesia and Surgical Critical Care, Hospital Clínico Universitario de Valencia, Valencia, Spain (Fco.Javier.Belda@uv.es); Borghi B: Department Modul of Research in Anaesthesia, IRCCS Rizzoli Orthopaedic Institute, Bologna, Italy (battista.borghi@ior.it); Rosencher N: Department of Anaesthesia and Surgical Intensive Care, Groupe Hospitalier Cochin - Saint Vincent de Paul, Paris, France (nadia.rosencher@cch.aphp.fr); Arnold G: Department of Anaesthesia, Imperial College NHS Trust, London, UK (Glenn.Arnold@imperial.nhs.uk); Albaladejo P: Department of Anaesthesia, Centre Hospitalier et Universitaire de Grenoble, Hôpital Michallon, La Tronche, France (palbaledejo@chu-grenoble.fr); Capdevila X: Department of Anaesthesia, Centre Hospitalier Régional Universitaire de Montpellier, Hôpital Lapeyronie, Montpellier, France (x-capdevila@chu-montpellier.fr); Minville V: Department of Anaesthesia and Intensive Care Medicine, Centre Hospitalier et Universitaire de Toulouse, Hôpital Rangueil, Toulouse, France (minville.v@chu-toulouse.fr); Kienbaum P: Department of Anaesthesia, University Hospital Düsseldorf, Düsseldorf, Germany (Peter.Kienbaum@med.uni-duesseldorf.de); Kunitz O: Department of Anaesthesia, Emergency and Intensive Care Medicine, Klinikum Mutterhaus der Borromäerinnen gGmbH, Trier, Germany (Oliver.Kunitz@mutterhaus.de); Mynard V, Chong CF: Air Liquide Santé International, Medical Gases Groupe, Claude Delorme Research Center, Jouy-en-Josas, France (Vanessa.Mynard@AirLiquide.com, Chui-Fung.Chong@AirLiquide.com).

## Supplementary Material

Additional file 1 **Confusion Assessment Method (CAM) questionnaire**[20]. Click here for file

Additional file 2 **Table S1. Sequental Organ Failure Score (SOFA)**[21,22]. Click here for file

Additional file 3 **Table S2. Modified Aldrete Score**[23]. Click here for file

Additional file 4 **The 11-point verbal rating scale**[24]. Click here for file
