# Supplementary material for: The Hip Fracture Surgery in Elderly Patients (HIPELD) study: protocol for a randomized, multicenter controlled trial evaluating the effect of xenon on postoperative delirium in older patients undergoing hip fracture surgery
Source: Trials. 2012 Sep 27;13:180. doi: 10.1186/1745-6215-13-180 (PMC3488510; doi:10.1186/1745-6215-13-180)
Supplement: Additional file 1 — Confusion Assessment Method (CAM) questionnaire[20]. [file 1745-6215-13-180-S1.docx]

**Appendix 1.**

Confusion Assessment Method questionnaire (adapted from Inouye SK et al. [27]):

I- Acute Onset and Fluctuating Course
a) Is there evidence of an acute change in mental status from the patient's baseline?

No/ Yes

b) Did the (abnormal) behavior fluctuate during the past day, that is tend to come and go or increase and decrease in severity?

No/ Yes

II- Inattention
Does the patient have difficulty focusing attention, for example, being easily distractible or having difficulty keeping track or what was being said?

No/ Yes

III- Disorganised Thinking
Was the patient’s thinking disorganized or incoherent, such as rambling or irrelevant conversation, unclear or illogical flow of ideas, or unpredictable switching from subject to subject?

No/ Yes

IV- Altered Level of Consciousness
Overall, how would you rate the patient’s level of consciousness?

- Alert (normal)

- Vigilant (hyperalert)

- Lethargic (drowsy, easily aroused)

- Stupor (difficult to arouse)

- Coma (unarousable)

Do any checks appear in this box ? No / Yes
