# Supplementary material for: The Hip Fracture Surgery in Elderly Patients (HIPELD) study: protocol for a randomized, multicenter controlled trial evaluating the effect of xenon on postoperative delirium in older patients undergoing hip fracture surgery
Source: Trials. 2012 Sep 27;13:180. doi: 10.1186/1745-6215-13-180 (PMC3488510; doi:10.1186/1745-6215-13-180)
Supplement: Additional file 2 — Table S1. Sequental Organ Failure Score (SOFA)[21,22]. [file 1745-6215-13-180-S2.docx]

**Appendix 3.**

Sequental Organ Failure Score: The scoring below is based on values described by Vincent JL et al. [29], with derivation of SpO_2_/FiO_2_ values corresponding to PaO_2_/FiO_2_ ratios described by Pandharipande PP et al. [30].

| Sequential Organ Failure Score | Respiratory  SpO_2_/FiO_2_ Ratio | Cardiovascular  Mean Arterial Pressure/Vasopressors^a^ | Nervous Glasgow Coma Score | Hepatic Bilirubin (µmol/L) | Renal Creatinine (µmol/L) | Coagulation Platelets (x10^3^/mm^3^) |
| --- | --- | --- | --- | --- | --- | --- |
| 0 | >512 | No hypotension | 15 | <20 | <110 | >150 |
| 1 | ≤512 | <70 mmHg | 13-14 | 20-32 | 110-170 | ≤150 |
| 2 | ≤357 | Dopamine ≤ 5 or dobutamine (any dose) | 10-12 | 33-101 | 171-299 | ≤100 |
| 3 | ≤214 | Dopamine > 5,  epinephrine ≤ 0.1 or norepinephrine ≤ 0.1 | 6-9 | 102-204 | 300-440 | ≤50 |
| 4 | ≤89 | Dopamine > 15, epinephrine > 0.1 or norepinephrine > 0.1 | <6 | >204 | >440 | ≤20 |

^a^ Adrenergic agents administered for at least 1 hour (doses given are in µg/kg/min).
