# Supplementary material for: The Hip Fracture Surgery in Elderly Patients (HIPELD) study: protocol for a randomized, multicenter controlled trial evaluating the effect of xenon on postoperative delirium in older patients undergoing hip fracture surgery
Source: Trials. 2012 Sep 27;13:180. doi: 10.1186/1745-6215-13-180 (PMC3488510; doi:10.1186/1745-6215-13-180)
Supplement: Additional file 3 — Table S2. Modified Aldrete Score[23]. [file 1745-6215-13-180-S3.docx]

**Appendix 4**

Aldrete Score modified according to Aldrete JA. A patient is considered ready to be discharged from the PACU when his/her Aldrete score reaches a value ≥ 9 [31].

| **Parameter** | **Description of patient** | **Score** | **Admission** | **30 minutes** | **60 minutes** |
| --- | --- | --- | --- | --- | --- |
| Activity Level | Moves 4 extremities voluntarily or on command | **2** |  |  |  |
|  | Moves 2 extremities voluntarily or on command | **1** |  |  |  |
|  | Cannot move extremities | **0** |  |  |  |
| Respirations | Breathes deeply and coughs freely | **2** |  |  |  |
|  | Is dyspneic, with shallow, limited breathing | **1** |  |  |  |
|  | Is apneic = 0 | **0** |  |  |  |
| Circulation (Blood Pressure) | *BP ± 20% of pre-anesthetic level | **2** |  |  |  |
|  | *BP ± 20% to 49% of pre‑anesthetic level | **1** |  |  |  |
|  | *BP ± 50% of pre-anesthetic level | **0** |  |  |  |
| Consciousness | Is fully awake | **2** |  |  |  |
|  | Is arousable on calling | **1** |  |  |  |
|  | Is not responding | **0** |  |  |  |
| Oxygen Saturation as determined by pulse oxymetrie | Has level >90% when breathing room air | **2** |  |  |  |
|  | Requires supplemental oxygen to maintain level > 90% | **1** |  |  |  |
|  | Has level < 90% with oxygen supplementation | **0** |  |  |  |
|  | **Total** |  |  |  |  |

*BP = Blood Pressure
