# Supplementary material for: The Hip Fracture Surgery in Elderly Patients (HIPELD) study: protocol for a randomized, multicenter controlled trial evaluating the effect of xenon on postoperative delirium in older patients undergoing hip fracture surgery
Source: Trials. 2012 Sep 27;13:180. doi: 10.1186/1745-6215-13-180 (PMC3488510; doi:10.1186/1745-6215-13-180)
Supplement: Additional file 4 — The 11-point verbal rating scale[24]. [file 1745-6215-13-180-S4.docx]

**Appendix 2.**

11-Point Verbal Rating scale adapted from Apfel CC et al. [28].

The patient will be asked to rate his/her nausea since the preceding visit on a 11-point Verbal Rating Scale (VRS) for nausea, with 0 = "no nausea" and 10 = "nausea as worst it could be".

In addition, any episode of retching *(an attempt to vomit that is not productive of stomach contents)* and/or vomiting *(oral expulsion of stomach contents)* will be recorded and counted as separate episodes:

- Vomiting: Yes/ No

- Retching: Yes/ No
